# Hyperalignment of motor cortical areas based on motor imagery during action observation

**DOI:** 10.1038/s41598-020-62071-2

**Published:** 2020-03-24

**Authors:** Salim Al-Wasity, Stefan Vogt, Aleksandra Vuckovic, Frank E. Pollick

**Affiliations:** 1 School of Psychology, University of Glasgow, Glasgow, G12 8QB UK; 2 School of Engineering, University of Glasgow, Glasgow, G12 8QB UK; 3 College of Engineering, University of Wasit, Wasit, Iraq; 4 Department of Psychology, Lancaster University, Lancaster, LA1 4YF UK

**Keywords:** Data processing, Learning algorithms

## Abstract

Multivariate Pattern Analysis (MVPA) has grown in importance due to its capacity to use both coarse and fine scale patterns of brain activity. However, a major limitation of multivariate analysis is the difficulty of aligning features across brains, which makes MVPA a subject specific analysis. Recent work by Haxby *et al*. (2011) introduced a method called Hyperalignment that explored neural activity in ventral temporal cortex during object recognition and demonstrated the ability to align individual patterns of brain activity into a common high dimensional space to facilitate Between Subject Classification (BSC). Here we examined BSC based on Hyperalignment of motor cortex during a task of motor imagery of three natural actions (lift, knock and throw). To achieve this we collected brain activity during the combined tasks of action observation and motor imagery to a parametric action space containing 25 stick-figure blends of the three natural actions. From these responses we derived Hyperalignment transformation parameters that were used to map subjects’ representational spaces of the motor imagery task in the motor cortex into a common model representational space. Results showed that BSC of the neural response patterns based on Hyperalignment exceeded both BSC based on anatomical alignment as well as a standard Within Subject Classification (WSC) approach. We also found that results were sensitive to the order in which participants entered the Hyperalignment algorithm. These results demonstrate the effectiveness of Hyperalignment to align neural responses across subject in motor cortex to enable BSC of motor imagery.

## Introduction

Multivariate Pattern Analysis (MVPA) has evolved as an effective tool in the analysis of fMRI data, and its usefulness has been shown in its ability to decode the neural responses associated with a variety of different brain states, including for instance, low-level visual features in the early visual cortex^1,2^, auditory stimuli in the auditory cortex^3^, and motor actions in sensorimotor cortex^4–8^. A major limitation of MVPA (for review, see^9^) is that it requires a subject-dependent analysis which uses a new classifier model for each individual brain because the structure of neural activation varies across subjects. Accordingly, it has been shown that the classifier performance drops when predicting another subject’s responses^10,11^. One possible explanation for this drop in performance of classifiers for Between Subject Classification (BSC) might be idiosyncratic neural responses across subjects. Another possibility is that it arises from imperfections in aligning brains across subjects. BSC requires a spatial correspondence to align fine-scale topographies across subjects’ brains.

There are two alignment approaches: anatomical and functional alignment. Anatomical alignment methods define features either in volume or surface spaces to fit a template space. For instance, Talairach normalization^12^ performs a piecewise affine transformation to align the 3D volume of the brain to a template using anatomical landmarks, while surface-based alignment is an advanced method of anatomical alignment that matches the curvature of cortical surfaces across subjects^13,14^. In contrast, functional alignment maps the neural responses across brains. Conroy *et al*.^15,16^ aligned subjects’ cortical surfaces using functional connectivity of surface nodes, removing the requirements for temporal correspondence. Similarly, Sabuncu *et al*.^17^, performed functional alignment of the cortex across-subjects by using a rubber-sheet-like warping of cortical surfaces that maximised the between-subject correlation of time-locked activity during movie viewing. In addition, Nenning *et al*.^18^ proposed a functional alignment method based on a diffeomorphic registration of resting state features. Whilst these techniques establish a spatial correspondence across subjects, they still do not afford BSC accuracies approaching those of Within Subject Classification (WSC), due to inter-subject functional loci variability^19,20^.

As an alternative, Haxby and colleagues^10^ recently developed an anatomy free alignment algorithm called Hyperalignment to align subjects’ representational spaces of ventral temporal cortex into a high-dimensional common space. Hyperalignment parameters that map the individual voxel space into a high dimensional common space (and vice versa), were obtained based on brain responses while subjects watched a movie (‘Raiders of the Lost Ark’) at full length^10^. Hyperalignment uses Procrustean transformation iteratively^21^ to align the coarse and fine scale topographies by finding the optimal orthogonal transformation matrix that minimize the Euclidean distances between two set of response patterns. Haxby *et al*.^10^ applied the resulting subject-independent classifiers to data from two category perception experiments and found that the classification accuracy of the BSC obtained via Hyperalignment of ventral temporal cortex was equivalent to that of conventional WSC, thus confirming the validity of the common model space across a range of stimuli. Hyperalignment has been extended to regularized hyperalignment with a connection to Canonical Correlation Analysis^22^ and to joint Singular Value Decomposition hyperalignment with an improvement in computational speed^23^. Following on from this original work, two new algorithms, searchlight Hyperalignment and connectivity Hyperalignment have been developed to produce a common high-dimensional model of the whole cortex using either complex, dynamic audiovisual stimuli or resting state functional connectivity^24,25^. One promise of applying Hyperalignment is the ability to generate a subject-independent classifier to use in real-time fMRI decoded neurofeedback applications. Having such a classifier would reduce scanning and analysis time compared to employing a subject-dependent MVPA and could also build on normal brain activity instead of a patient’s potentially abnormal one^26,27^.

The main objective of our research was to assess whether Hyperalignment could successfully be applied to motor cortical areas, namely primary motor (M1) and premotor cortices (PM) to align the fine-scale structure associated with motor activation. Rather than asking participants to physically perform motor actions, we employed two well-researched forms of motor simulation^28^, namely action observation (AO) and motor imagery (MI). The neural substrates of motor execution, AO, and MI are partly overlapping^29,30^, and both AO and MI have been shown to facilitate motor learning (e.g., Higuchi *et al*.^31^). MI has also been shown to activate bilateral premotor cortex^29^. AO and MI have also been successfully applied in motor rehabilitation (see Vogt *et al*.^32^). In addition, WSC of individual actions has already been demonstrated for both observed and imagined actions^4–8^.

Extending Hyperalignment to motor cortical areas advances the earlier work on Hyperalignment that was limited to ventral temporal cortex^10^. While more recent work on Hyperalignment has examined ways to effectively extend the analysis to the whole brain, little has been reported about its ability to represent fine-scale structure related to motor control. For example, Guntupalli *et al*.^25^ applied searchlight Hyperalignment to the whole cortex and tested its effectiveness using Intersubject correlation (ISC) in watching a film (Raiders of the Lost Ark), but they did not examine BSC of any motor regions/tasks. Similarliy Guntupalli *et al*.^24^, derived a common model space based on functional connectivity of resting-state fMRI data in order to map task (emotion, gambling, language, motor, relational, social, and working memory) fMRI data taken from the HCP database^33,34^. However, as discussed by Guntupalli and colleagues^24^ this analysis did not reveal the fine-grained topography of these tasks as would be provided by dynamic stimuli.

Our fMRI experiment consisted of two sessions, where the first served to obtain hyperalignment parameters for the motor cortical areas and the second session was then used to contrast WSC and BSCs accuracies. This design allowed us to pursue the following aims:

1. To assess the effect of permutating the order in which participants were entered in the Hyperalignment procedure to generate a common model, in order to obtain the most discriminative subject-independent classifiers.
2. Based on the above, to validate the classification accuracy of BSC derived from Hyperalignment with (a) that derived from anatomically aligned data, and (b) with the accuracy of WSC where a new classifier is tailored for each subject.
3. To contrast the classification accuracy of two different ways of obtaining classifiers via Hyperalignment, (a) those obtained from a separate fMRI session (Session 1), and (b) those obtained within the same session (Session 2).
4. Finally, to assess the cost in classification accuracy when the to-be-classified participant’s data were excluded from the dataset used for the Hyperalignment (as to inform future applications of decoded neurofeedback where this scenario would be the norm).

## Methods

We obtained fMRI data from two separate recording sessions. In the first session, participants engaged in a combination of action observation and motor imagery (‘AO+MI’) of 25 action stimuli, as to obtain a rich sampling of neural responses for deriving a common model space. Whilst AO and MI have traditionally been studied in isolation and by different research groups, there is now growing evidence that both forms of motor simulation can be used concurrently, and that AO+MI generates both enhanced behavioural effects and more robust neural activation patterns than AO or MI alone (for review, see Vogt *et al*.^32^, Eaves *et al*.^35^). All stimuli used in Session 1 were stick figure displays that were blended, with varying weights, between three natural multijoint actions that anchored the space of blended movements (lifting, knocking, and throwing^36^). This set of blended movements are biomechanically possible actions that sample the space between the anchor points and thus provide a set of movements specially adapted for studying the anchor points.

In the second fMRI session, participants engaged in pure MI of the three natural (lifting, knocking, and throwing) actions that formed the basis of the above blend space. We used this independent dataset in order to contrast the classification accuracy of the subject-independent classifiers of the common model space derived from the Hyperalignment with the accuracy of classifiers based on conventional anatomically aligned data.

### Participants

Ten right-handed individuals (age 32.5 ± 7.6, 8 male) with normal or corrected to normal vision participated in the experiment. All subjects provided their informed consent for the experiment. The ethics committee of the College of Science and Engineering, University of Glasgow provided ethical approval. All methods were performed in accordance with the relevant guidelines and regulations provided by the ethics committee of the College of Science and Engineering

### Stimuli

**Figure 1:**
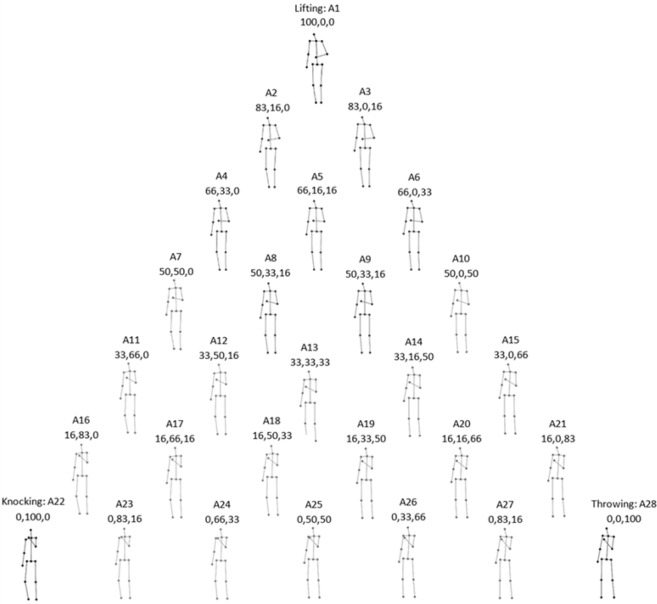
The stick-figures displaying the 60^th^ frame of each action along with the blending weights (the percentage change of lifting, knocking and throwing respectively)^50^.

Stimuli consisted of a set of 28 movies that depicted the human body as a stick figure, with point-lights at the major joints, performing arm actions^36^. All movies had a duration of 2000ms with a total of 120 frames shown at 60 Hz and with a resolution of 256 × 256 pixels. Three of the 28 movies were the 3 actions of lifting, knocking and throwing, subsequently referred to as the ‘natural actions’. The remaining 25 movies were 2-way and 3-way blends of these 3 actions (Fig. 1), subsequently referred to as the ‘blended actions’. The blended actions were made using the algorithm of Kovar and Gleicher^37^, which preserves the constraints of biomechanical movement. They were created in a step size of 16.6% change of the weighting of the original actions producing 15, 2-way blended actions (Fig. 1, outer triangle) and 10, 3-way blended actions (Fig. 1, inner triangle and center). These blended stimuli were chosen since they finely sample the space around our target actions and thus provide a useful input for the hyperalignment algorithm to represent the three different arm movements.

### Apparatus and procedure

Each participant attended two different scanning sessions. The stimuli were displayed via a LCD projector onto a rear projection screen that was viewed through a mirror mounted on the head coil. Videos subtended approximately 2.6 horizontal and 8.8 vertical degrees of visual angle. Stimulus presentation was controlled using Presentation software (Neurobehavioral Systems, Inc.).

#### Session 1: Blended action stimuli

**Figure 2:**
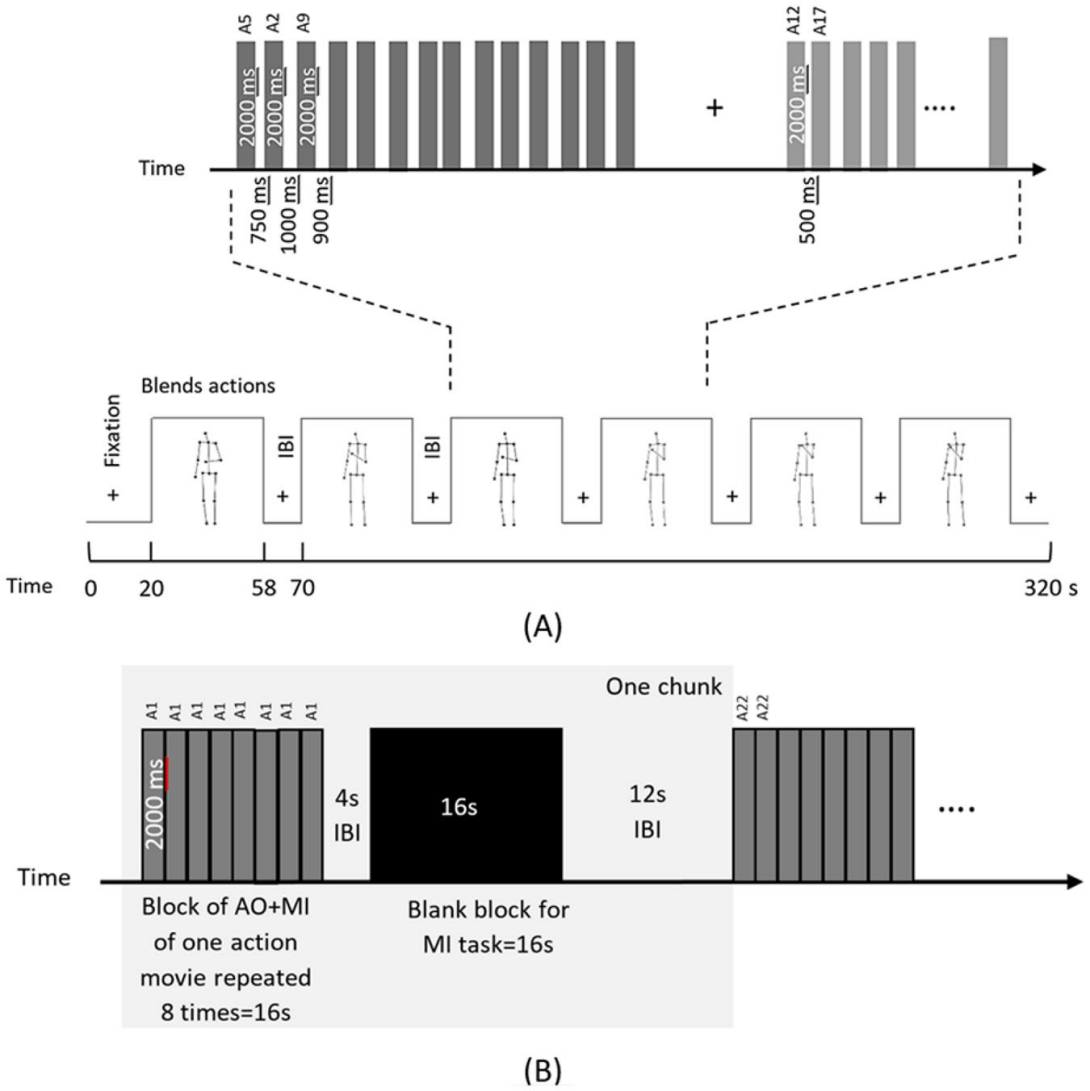
(**A**) Experimental design of blended actions session (Session 1). Each run consisted of 6 blocks of 38 s with IBI of 12 s. After 20 s fixation, subjects started to simultaneously perform action observation and motor imagery (AO+MI). In each block, 14 video trials of 2000ms each were presented with a variable ISI from 500–1000 ms. (**B**) Experimental block design of natural actions session (Session 2). Each run contained 9 chunks (gray shaded area), and there were 3 chunks for each action. A chunk comprised an AO+MI block with 8 repetition of the same action video of 16 s, a short IBI of 4s, a pure MI block of 16 s and a long IBI of 12 s. After 20s fixation, participants observed and imagined (AO+MI) doing an action simultaneously for 8 times then imagined (MI) it 8 times assisted by a pacing tone^50^.

In Session 1, subjects were presented with the 25 movies of the blended actions and engaged in *concurrent* action observation and motor imagery (AO+MI) involving their right arms. The movies were presented in 10 separate runs of 320 s each using a block design. Each run started with 20s fixation and contained 6 blocks of stimuli with 12 s of inter-block interval (IBI). Within a block, 14 pseudorandomly chosen actions were presented with a variable interstimulus interval (ISI) that ranged from 500 ms to 1000 ms pseudorandomly as shown in Fig. 2(A). As a catch trial to enforce attention, subjects performed a fixation color change task by pressing a response button with their left hand when the fixation color changed from black to red. All subjects observed the same sequence of the runs, blocks and trials.

#### Session 2: Natural action stimuli

In Session 2, only the three natural action displays (lifting, throwing and knocking) were used. In this session, each block of pure MI tasks was preceded by a block of tasks involving action observation with motor imagery (AO+MI). The AO+MI blocks primarily served to remind participants on the kinematic details of each action. Whilst the methodological focus of the present paper was on the pure MI condition of Session 2 as an independent key dataset, for completeness the results for the AO+MI condition of Session 2 are provided in Supplementary Materials 1. In this session, subjects were instructed to either imagine or observe and imagine moving their right arm. During the pure MI condition, participants were prompted by a pacing tone that was presented over MRI-compatible headphones and controlled via Presentation software (Neurobehavioral Systems, Inc.). The auditory pacing signal consisted of beeps (100 ms duration at 500 Hz) repeated every 2 s. Subjects were instructed to synchronize their MI with that signal. The videos were shown in a block design of 8 runs, 456s each. Each run started with 20s fixation at the beginning and consisted of 9 chunks, with each natural action presented 3 times randomly. Chunks were randomized across participants. A chunk consisted of a block of AO+MI, a short IBI of 4s, and a block of pure MI followed by a long IBI of 12s. Each block lasted for 16 s and either had 8 videos of the same action for the case of AO+MI or a black screen for the case of MI, as shown in Fig. 2(B). A catch trial was included where subjects performed a fixation color change task.

### Imaging protocol

Subjects were scanned in a 3 Tesla Siemens Tim Trio MRI scanner at the University of Glasgow Centre for Cognitive Neuroimaging (CCNi) with a 32-channel head coil. T1 weighted structural scans were acquired at the middle of each session (TR = 2300 ms, TE = 2.96 ms, 192 sagittal slices, 1 mm isotropic voxels and image resolution 256 × 256). T2*-weighted functional scans were collected with an Echo Plannar Imaging (EPI) sequence (TR = 2000ms, TE = 30 ms, whole brain coverage with 32 axial slices, with 0.3 mm gap, 3 mm isotropic voxel).

### fMRI data preprocessing

Data were preprocessed using BrainVoyager QX 2.8.4 (Brain Innovation, Maastricht, The Netherlands). The anatomical data (T1) processing comprised Background cleaning, Brain extraction and intensity inhomogeneity correction. Functional data (T2*) preprocessing involved slice scan-time correction with cubic-spline interpolation, 3D motion correction with Trilinear/Sinc interpolation, linear trend removal and high-pass filtering with a cutoff set to 2 cycles. All the functional volumes of each session were aligned to a reference volume, which was the first functional volume after the anatomical scan. The functional data of each subject of both sessions were spatially aligned to a common anatomical space to establish a voxel correspondence across sessions and then normalized to Talairach space^12^.

### fMRI data analysis

All further fMRI data were analyzed using MATLAB (version 9.0, Mathworks), NeuroElf v1.0 (www.neuroelf.net) and PyMVPA 2-2.6.0 (^38^ www.pymvpa.org), except for an exploratory univariate analysis of the Session 2 data, see Supplementary Materials 4.

#### Anatomical-based voxel selection

A gray matter mask, derived using a Talairach brain atlas available in BrainVoyager, was used to extract data from Brodmann areas 4 and 6 of the left hemisphere. Only left hemisphere data were chosen since the Hyperalignment process is more appropriate for focused brain regions. The resultant mask had 1758 3 mm isotropic voxels. The time-series of activation for each voxel was normalized using z-score and shifted by 2 TRs (4s) to account for the hemodynamic delay^39–41^.

#### Building a common model representational space using hyperalignment

**Figure 3:**
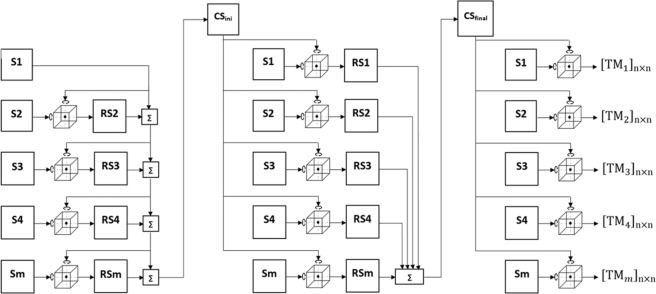
A block diagram of the Hyperalignment method used to derive a common representational space and transformation parameters (TM) using Procrustean transformation. S is the subject’s representational space, RS is the subject’s aligned (rotated) space using procrustean transformation, CS_ini_ is the intermediate common spaces, CS_final_ is the final common space and ∑ is the average of representational spaces^50^.

Brain activation patterns can be considered as vectors in a high-dimensional space in which each dimension is a feature (fMRI voxel). Although the space of individual subjects are not aligned, they are time locked to the stimuli^10^. Hyperalignment uses Procrustean transformation iteratively to map subjects’ representational spaces into a common model space by deriving transformation parameters for each subject. This process consists of three stages: during the first stage, an arbitrary subject is chosen to be a reference subject. The 2nd subject’s response vectors are aligned to the reference subject’s vectors using Procrustean transformation and then the 3rd subject is aligned to the mean response vectors of the first 2 subjects and so on. In the second stage, each subject’s response vector are aligned to the mean response vectors of the first stage (intermediate common space) and new response vectors are computed by averaging all the subjects’ aligned (rotated) vectors which would be the final common model space. During the last stage, Hyperalignment transformation parameters are calculated for each subject to map their voxel space into the final common model space as shown in Fig. 3. This common model space and transformation parameters were derived using the response vectors of all subjects during the AO+MI task of the blended actions stimuli session.

#### Mapping the subjects’ representational space into the common model space

Subjects’ representational spaces of the MI task obtained during the natural actions session can be aligned altogether by multiplying each subject’s transformation parameters with the voxels’ responses as illustrated in Eq. 1.

1 $${M}_{(k\times n)}={V}_{(k\times n)}{T}_{(n\times n)}$$

Where *M*_*(k×n)*_ is the mapped matrix in the common model space (*k* time points, *n* voxels), *V*_*(k×n)*_ is the voxels’ responses in Talairach space and *T*_*(n×n)*_ is the Hyperalignment transformation parameters.

#### Multi-Voxel pattern analysis

For the classification (lifting vs. knocking vs. throwing) of the brain activity during the MI task a multiclass linear Support Vector Machine (SVM)^42^ implemented in PyMVPA, with a penalty constant (*C*) of negative value was used. The latter provided an automatic scaling of its value according to the norm of the training data. The multiclass problem was translated into a series of binary classifications paired with a majority voting scheme to determine the predicted class^10,43,44^. For within subject classification (WSC), a leave-one-run-out approach was used, which resulted in an 8 fold cross-validation. A leave-one-subject-and-one-run-out scheme was used for between subject classification (BSC), which generated an 80 folds cross-validation across runs and subjects. For each scenario (BSCs and WSC), we then computed averages across folds accordingly. For each modality, data within similar stimulus blocks of each run were averaged to substitute the response patterns for that stimulus, such that 3 samples correspond to the three action stimuli per run. For each scenario individually (WSC, BSC based on anatomical alignment and BSC based on hyperalignment), a SVM searchlight approach^45^ with a sphere radius of 3 voxels was applied on session 2 data and within the anatomically defined mask to define the network of regions that had information to decode the three stimuli, and the accuracies of searchlight maps were mapped onto a cortical surface for visualization. A set of top performing voxels from the searchlight was chosen to serve as a final ROI for subsequent MVPA analyses. The criterion for defining this set of voxels was determined by examining performance across increasing voxel set sizes and choosing the number of voxels where classification accuracy reached asymptote. WSC was performed by training the SVM classifier on the data from seven runs (7 runs × 3 stimuli samples = 21 pattern vectors) and tested on the left-out eighth run (3 pattern vectors) of each subject independently. For BSC, the SVM classifier was trained on the data from 1 run in 9 subjects (1 run × 9 subjects × 3 stimuli samples = 27 pattern vectors) and tested on the left-out run of the left-out subject (1 run × 1 subjects × 3 stimuli samples =3 pattern vectors). BSC was performed on the data that were aligned anatomically using Talairach space and on the data that were mapped in the common space using Hyperalignment. A 1-way mixed effect analysis of variance (ANOVA) was performed to compare the classification accuracies between WSC, BSC based on anatomical alignment and BSC based on Hyperalignment.

#### Common model space generalization

The common model space was derived by averaging all the subjects’ rotated representational spaces during the second stages of Hyperalignment (see Fig. 3). To test the generalization of this model for a new representational space that had not played any role in deriving it, a leave one subject out of Hyperalignment procedure was performed to create a common model space and then to map the left-out subject to that space and derive the transformation parameters. BSC based on Hyperalignment was conducted by training the SVM classifier on the Hyperaligned data of 9 subjects and tested using the newly mapped subject data. A 1-way mixed effect analysis of variance (ANOVA) was run to compare the classification accuracies between different Hyperalignment procedures.

#### Common model space sensitivity

To compute the common model representational space a reference subject is chosen arbitrarily (when the number of voxels are equal across subjects) during the first stage of Hyperalignment and the rest of the subjects contribute by different weights (see Fig. 3), To test the sensitivity of the common model space to the choice of reference subject and subject order, the subject order was shuffled 2000 times prior to calculating Hyperalignment and for each time a new common model space and transformation parameters were computed to map subjects’ activation patterns of the MI task and then to predict the MI class (lift, knock or throw).

### Ethical approval

All procedures performed in studies involving healthy human participants were in accordance with the ethical standards of College of Science and Engineering, University of Glasgow.

### Informed consent

Informed consent was obtained from all individual participants included in the study.

## Results

The results of the two fMRI recording sessions are reported. In Session 1, 10 subjects performed an AO+MI task to presentation of 25 videos displaying a parametric set of action blends of lifting, knocking and throwing. These data were used to compute Hyperalignment transformation parameters that allowed us to transform the data of individual subjects into a common model space. In Session 2, the same subjects performed pure MI to presentation of 3 videos displaying the actions lifting, knocking and throwing. From these data, SVM classifiers were used to decode the MI conditions of each individual subject using that subject’s anatomically aligned data and hyperaligned data (in Supplementary Materials 1, we show the equivalent analysis applied to the AO+MI dataset of Session 2). In the following, we first explore the sensitivity of the common model space to the subject order used to estimate Hyperalignment parameters (aim 1). Following this, we use the subject order that produced the highest performance of the common model space and map all the subjects’ MI data to it. Then we compare between WSC, BSC based on anatomical alignment and BSC based on Hyperalignment using the blended actions for each modality separately (aim 2). Additionally, we compare between BSC based on Hyperalignment derived using the 25 blended actions and the 3 natural actions (aim 3). Finally, we examine the impact of a common model generalization on BSC (aim 4).

### Sensitivity of the common model space to subject order

**Figure 4:**
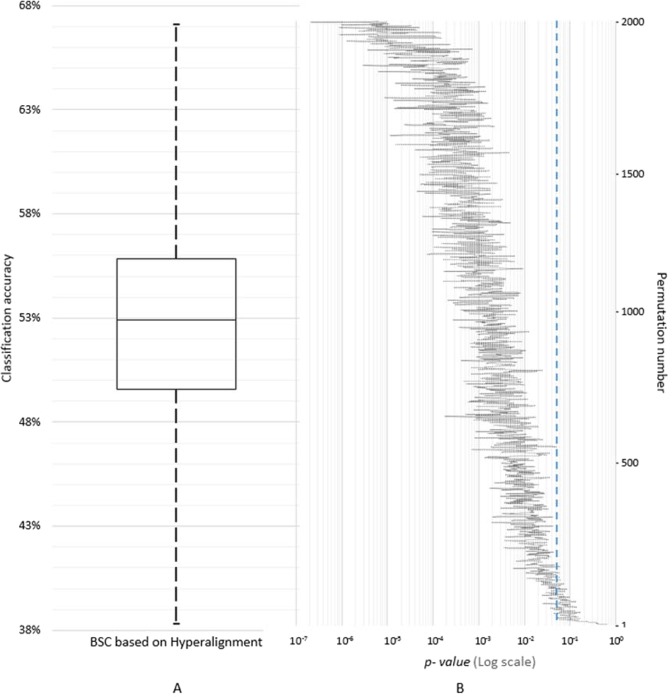
(**A**) A box-whisker plot of BSC based on Hyperalignment of 2000 permutations of subject order showing the classification accuracies of different orders of subjects, (**B**) logarithmic scale of two-tailed t-tests between BSC based on Hyperalignment and BSC based on Anatomical alignment, dashed line is *p* = 0.05, t(18) = 2.09^50^.

To assess the influence of the subject order on the common model space, 2000 permutations of subject order were performed to derive new common model spaces and Hyperalignment transformation parameters using the neural responses of the 25 blended actions. From this, the data of the MI task were mapped into the common model spaces and the distribution of BSC was estimated. Figure 4 presents the distribution of MI classification accuracies based on Hyperalignment using a box-whisker plot with a median classification accuracy of 52.9% and a maximum of 67% (Fig. 4, panel A; see Fig. S1 for the AO+MI data), along with logarithmic scaled two-tailed t-tests results between BSC based on Hyperalignment and Anatomical alignment (Fig. 4, panel B).

Given that Haxby *et al*.^10^ did not report an influence of subject order on Hyperalignment performance, we examined our data for possible explanations. One possibility would be that this effect is being driven by poor performance by one or more participant. While due to the difficulty of incorporating into our scanning paradigm we did not acquire any performance indices of motor imagery (e.g.^46,47^), we did incorporate the color-changing task to monitor attention to the task. Our data on the color changing task revealed that all participants were at ceiling of 82–86% correct to see if there was variability in performance task attention. We also examined head movements and subjects had less than 3 mm or 3 degrees of change in their head movement. In addition, since the first subjects into the Hyperalignment algorithm might unduly influence the result we examined whether there was a clear effect of particular subjects entering at the beginning of the Hyperalignment process. Results showed that the effect of order of the subjects entering the Hyperalignment algorithm is complex and not reliant simply on the first participant(s), as shown in Fig. S2.

### Between subject classification after hyperalignment based on brain response to the 25 blended actions

The common representational space and the Hyperalignment transformation parameters were derived using the neural responses of the 25 blended actions obtained while participants performed AO+MI. In this study, n = 130 3 mm isotropic voxels were selected using a Searchlight approach and were used as a ROI for all the subsequent analyses. BSC using Hyperalignment was performed using subjects’ response patterns on the MI task (lift, knock, and throw, respectively) that were mapped into the common space using the associated Hyperalignment parameters. As a comparison, BSC using anatomical alignment was also performed on the subjects’ data that were aligned based on anatomy using Talairach space^12^. BSC accuracies using both Hyperalignment and anatomical alignment were relatively stable over a wide range of voxels sets. As an additional step, BSC values were compared to WSC in which an individual classifier was constructed for each subject.

**Figure 5:**
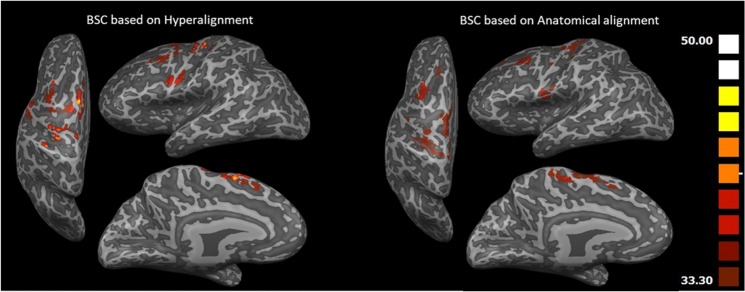
Searchlight maps showing classification accuracies of BSC based on Hyperalignment (left) and BSC based on anatomical alignment (right) using the MI task (lifting, throwing and knocking). Hyperalignment transformation parameters were derived using the responses to the blends action movies^50^.

Figure 5 shows the Searchlight maps of BSC based on Hyperalignment and BSC based on anatomical alignment. Due to Hyperalignment, the representational space of Searchlight spheres are well aligned with an overall accuracy above 33.3% (chance level) using a common model space with a peak of (47.5%) in Supplementary Motor Area as well as M1 and dorsal PM (PMd) regions in contrast to anatomical alignment with a peak of (38.75%) in M1.

**Figure 6:**
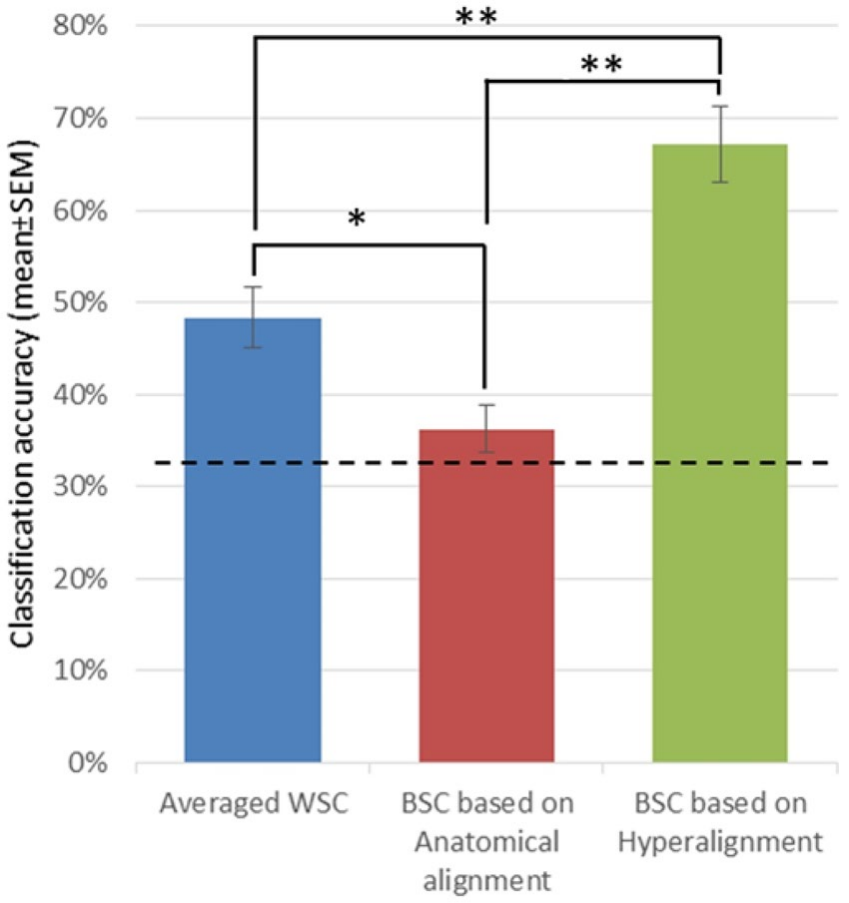
Classification accuracies (mean ± SEM) for averaged WSC, BSC based on Anatomical alignment and BSC based on Hyperalignment, dashed line indicates chance level (33%). *P ≤ 0.05, **P ≤ *0.0001*^50^.

BSC accuracy of anatomically aligned data was (36.26%, SE = 2.5%, chance = 33%). In contrast, after the Hyperalignment, BSC predicted the three actions with a substantially enhanced accuracy of 67.08% (SE = 4.1%). On the other hand, the average WSC accuracy (48.3%, SE = 3.29%) was well below than the BSC of Hyperaligned data as shown in Fig. 6 (and Fig. S3 for the AO+MI dataset, with equivalent results). A one-way repeated measures ANOVA using Greenhouse-Geisser correction indicated a statistically significant difference between approaches, *F*(1.504,13.532) = 36.05, *p* < 0.0001. Bonferroni post hoc tests indicated that the classification accuracy for BSC based on Hyperalignment (67.1%), BSC based on anatomical alignment (37.1%) and WSC (48.3%), were significantly different from each other (*ps* < 0.019).

**Figure 7:**
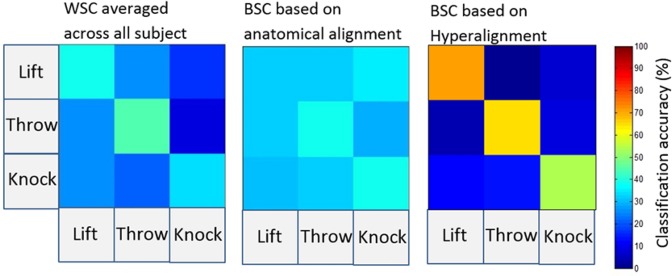
Confusion matrices for action classification using WSC, BSC based anatomical alignment and BSC based on Hyperalignment^50^.

The confusion matrix of BSC based on Hyperalignment shows that the classifier identifies the individual actions as shown in Fig. 7, on the contrary, BSC based anatomical alignment does not show any discrimination among actions.

### Between subject classification after hyperalignment based on brain response to the natural actions

The common representational space and the Hyperalignment transformation parameters can be derived from other sources of fMRI data^10^. Thus, in addition to the common representational space and Hyperalignment transformation parameters based on neural responses of the AO+MI task on the blended actions we ran another analysis to investigate whether the Hyperalignment parameters derived using only the neural responses of the MI task on the three natural actions would afford a high BSC accuracy. The common space was derived using all runs minus one. Then the left-out run of each subject was mapped into the common space using the Hyperalignment parameters obtained earlier. To avoid double dipping^48^, a classifier was trained on the left-out runs of all the subjects except one and was tested on the data of the left-out run in the left-out subject and hence, the test data play no role either in training the classifier or in Hyperalignment. BSC accuracy after Hyperalignment based on the data of the same session was almost identical to Hyperalignment based on the blended action movies (69.17%, SE = 2.42% versus 67.08%, SE = 4.1%, respectively.

### Common model space generalization

**Figure 8:**
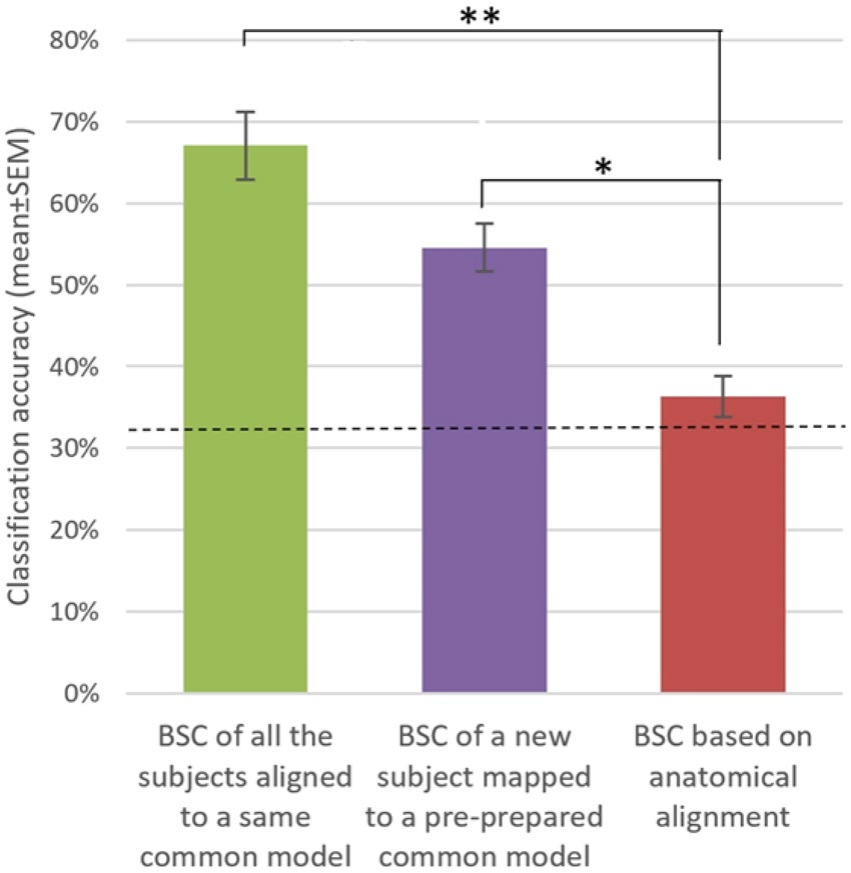
BSC based on Hyperalignment of a common model space derived using all the subjects’ data or derived using all the subject except one. Dashed line is chance level = 33%. *P ≤ *0.01*, **P ≤ *0.0001*^50^.

We conducted a final analysis to study the decoding effect of aligning new subjects to the common model space who have not contributed to derive it. Figure 8 shows the BSC based on Hyperalignment of a common model derived using either all the subjects’ data or by mapping a new subject to a pre-prepared common model. Again, the one-way repeated measures ANOVA with a Greenhouse-Geisser correction revealed a significant difference in classification accuracies between the BSC approaches, *F*(1.899,17.09) = 25.298, *p* < 0.0001. Specifically, the BSC based on anatomical alignment (37.1%) was significantly lower compared to BSC based on Hyperalignment derived from all subjects (67.1%, *p* < 0.0001) and compared to BSC based on Hyperaligment derived from all subject-1 (54.6%, *p* < 0.003). The difference between BSC based on Hyperalignment derived from all subjects and BSC based on Hyperaligment derived from all subject-1 just failed the conventional significance level (p = 0.063). Thus, although engaging all the subjects’ representational spaces fine tunes the common model space, aligning a new subject’s representational space to a pre-defined common model still shows benefit over BSC based on anatomical alignment. This property could prove beneficial during paradigms using online fMRI classification.

## Discussion

In this paper, we performed Hyperalignment to map the functional voxel spaces of the activity in individual subjects’ motor regions into a common model space. Hyperalignment uses Procrustean transformation to rotate subject’s representational axes into a common coordinate system in which the neural responses for the same stimuli or tasks are in optimal alignment across individuals. This model is a high-dimensional representational space that captures the fine-scale topographies and their variability across brains. Hyperalignment computes the transformation parameters for each subject that can be applied to map a different set of response vectors using the same subjects’ voxels, into a common model space. Previously, Haxby and colleagues have explored the feasibility of using Hyperalignment to align fMRI data in visual and auditory cortices^10,22,23,25^ however it is unknown whether the technique works to align other brain regions such as sensorimotor regions. Here, we used Hyperalignment to compute a common model representational space of the motor cortical areas from data obtained while participants performed an AO+MI task, and we validated that model by decoding the brain activity during MI of different arm actions.

The first aim of our research was to explore whether the order in which participants were entered into the Hyperalignment procedure mattered for the generation of a discriminative subject-independent classifier. Previous reports of Hyperalignment results did not provide any analysis of subject order^10^, however the Hyperalignment algorithm is potentially sensitive to subject order. The inter-subject response variability to the task and different weights of subject’s contribution toward computing the intermediate common model (see Fig. 3) influence the Hyperalignment algorithm in its ability to rotate and align response vectors of the same stimulus across subjects, as the first subject entering the hyperalignment procedure will be serving as an initial common model space for the subsequent alignments and the last subject will be contributing by 50% toward creating the intermediate common model space. The results of our permutation test that shuffled the subject order and computed a new common model in each iteration showed that subject order had a profound effect. Why subject order had a substantial effect for our study exploring motor cortical regions is an area deserving further research. To be effective the common model representational space must be capable of capturing the fine-grained representation structures encoded in subjects’ responses. It is well possible that the premotor and motor regions, as analysed here, provide *per se* more inter-subject response variability for the representation of actions than obtained in the representation of objects in temporal cortex^10^. Another possible source of differential variability is that Haxby and colleagues^10^ used a large range of natural dynamic audiovisual stimuli (i.e., watching a movie), whilst we used a set of blended movements that densely sampled a particular movement space. Thus, independent of the anatomical region of interest, it is also possible that the wider range of stimulation used by Haxby and colleagues might have produced, overall, a less variable cross-participant response pattern than the more narrow stimuli provided by the blended lifting, knocking, and throwing movements used in the present study.

Third, one could argue that the AO+MI task used here might have produced higher inter-subject variability then Haxby and colleagues’ movie observation task. However, to the best of our knowledge, concurrent engagement in action observation and motor imagery is a highly efficient task in order to maximise the involvement of motor cortical regions without engaging overt movement^32,35^, and this was indeed the case in the present study where univariate analysis showed greater activity in BA6 for motor imagery of actions compared to baseline (see Supplementary Materials 4). Our analysis was based on activation patterns in premotor and primary motor cortex (BA6 and BA4). Regarding premotor regions, the most encompassing meta-analysis to date^29^ clearly indicates the involvement of bilateral premotor cortices in motor imagery. In addition, Pilgramm *et al*.^5^ and Zabicki *et al*.^4^ demonstrated that different imagined actions could be decoded successfully from the activation patterns in premotor and posterior parietal cortices. Regarding the recruitment of primary motor cortex during motor imagery, the available activation studies yielded more mixed results. In one of the few studies using multivariate analysis techniques, Sharma and Baron^49^ found indeed independent components shared between motor imagery and execution in dorsal premotor cortex as well as BA4. In summary, amongst the possibilities outlined above, future research would be required to identify the specific reason(s) why subject order had a substantial effect in the present study but not in previous research. For example, using suitable motor execution tasks instead of the AO+MI task chosen in the present study, might reduce inter-subject variability and thus subject order effects.

Based on the above, the second (and core) aim of this study was to compare the classification accuracy of BSC derived from Hyperalignment with BSC using anatomical alignment, and with the more standard WSC approach, using a separate dataset that involved a MI task. Importantly, the results demonstrated that mapping from individuals’ voxel spaces into a high dimensional common space, which captured the coarse and fine-scale representation structures encoded in subjects’ responses, afforded a BSC that significantly exceeded the BSC based on anatomical alignment. In addition, the standard individualised WSC-based classification accuracy exceeded that of BSC with anatomical alignment, but the former was still significantly lower than BSC based on Hyperalignment (see Fig. 6) due to the variability of individual subjects’ MI performance and therefore the overall classification accuracy. The average WSC of the MI task in left motor cortex was comparable to that reported in the studies by Zabicki *et al*.^4^ and Pilgramm *et al*.^5^. Interestingly, our paradigm involved imagining moving the whole right arm (complex actions) while the above two studies used simple actions that only engaged the fingers of the right hand. Our findings illustrate that Hyperalignment offers higher classification accuracies by building a common model of the motor cortex to increase the power of MVPA to detect the fine scale information across participants.

It is worth noting that a limitation of our current design is that all our MI blocks in Session 2 were preceded by an AO+MI block to instruct the action where the IBI was only 4s. Thus, the response of our MI block would have been influenced by the AO+MI response and therefore our results should not be interpreted as hard evidence of crossmodal transfer from AO+MI-derived hyperalignment to MI. However, aim 2 was not specifically to demonstrate crossmodal transfer. Rather, the result that Hyperalignment parameters, derived from the blended movements in Session 1, enhanced the classification of an independent fMRI dataset indeed satisfied this aim.

Whilst in the above validations, Hyperalignment parameters were derived from Session 1 and applied to Session 2, in principle Hyperalignment parameters can be computed from any fMRI data^10^, and it is thus not clear what limits there are for creating valid classifiers. Accordingly, our third aim was to contrast two different classifications based on Hyperalignment, one based on the above model, and the other using the fMRI signal from the MI task of all runs except one of the subjects. Indeed, our results showed that both models afforded comparable levels of classification accuracy. These results further demonstrate the flexibility of Hyperalignment to align subjects’ representational spaces using different data sources.

Finally (aim 4), we were interested in whether Hyperalignment would be successful in the situation when data from a subject not used in the original Hyperalignment creation process was encountered. To explore this we examined the cost in classification accuracy when a to-be-classified participant’s data were excluded from the dataset used for the Hyperalignment. Although there was a decrease in performance, classification accuracy was still better than BSC based on anatomical alignment. Such a situation would arise if Hyperalignment based on one set of participants would be used to perform classification on data from a new subject and could be expected in applications like real-time decoded neurofeedback. A new subject would require a mapping of their neural responses into a previously computed common model, and an estimate of their transformation parameters based on neural responses to the original data set could be used to obtain the transformation parameters. The computed parameters would be used to transform each new subject’s data into the common model space and subsequently, feedback would be given by a pre-trained classifier that identifies the brain states of the new subject Hyperaligned data.

In conclusion, we introduce here a common high dimensional representational model of the motor cortex in order to align the fine-grained structure of brain responses during MI task of natural actions^50^. This model is derived by using a Hyperalignment algorithm that rotates subjects’ representational spaces into one common space, affording high BSC accuracies compared to BSC based on anatomical landmarks. Hyperalignment can be used to align data from similar experiments into one common model that establishes a functional correspondence of fine-scale topographies across brains, enabling the use of these datasets to examine neural representations and individual differences. More importantly for MVPA analysis, this common model would reduce the time and cost of scanning by avoiding collecting extra data to train classifiers.

## Supplementary information

Supplementary material.
