# Supplementary material for: Hyperalignment of motor cortical areas based on motor imagery during action observation
Source: Sci Rep. 2020 Mar 24;10:5362. doi: 10.1038/s41598-020-62071-2 (PMC7093515; doi:10.1038/s41598-020-62071-2)
Supplement: Supplementary file 1 — Supplementary material. [file 41598_2020_62071_MOESM1_ESM.pdf]

Title: Hyperalignment of motor cortical areas based on motor imagery during action observation

Author names and affiliations:

Salim Al-Wasity[1,2,3*], Stefan Vogt[4], Aleksandra Vuckovic[2] and Frank E. Pollick[1]

[1]School of Psychology, University of Glasgow, Glasgow G12 8QB, UK

[2]School of Engineering, University of Glasgow, Glasgow G12 8QB, UK

[3]College of Engineering, University of Wasit, Wasit, Iraq

[4]Department of Psychology, Lancaster University, Lancaster LA1 4YF, UK

Supplementary material

1. Sensitivity of the common model space to subject order during AOMI and MI

In our main analysis, the common model spaces created in section 2.6.6 were used to individually map the MI data of Session 2 into the common model spaces, and the distribution of BSC was estimated using classifiers trained and tested on the MI dataset of Session 2. To supplement this analysis, here we report the results of the same procedure applied to the AOMI data of Session 2. As shown in Figure S1, for the AOMI dataset classification accuracies are marginally higher than for the MI dataset.

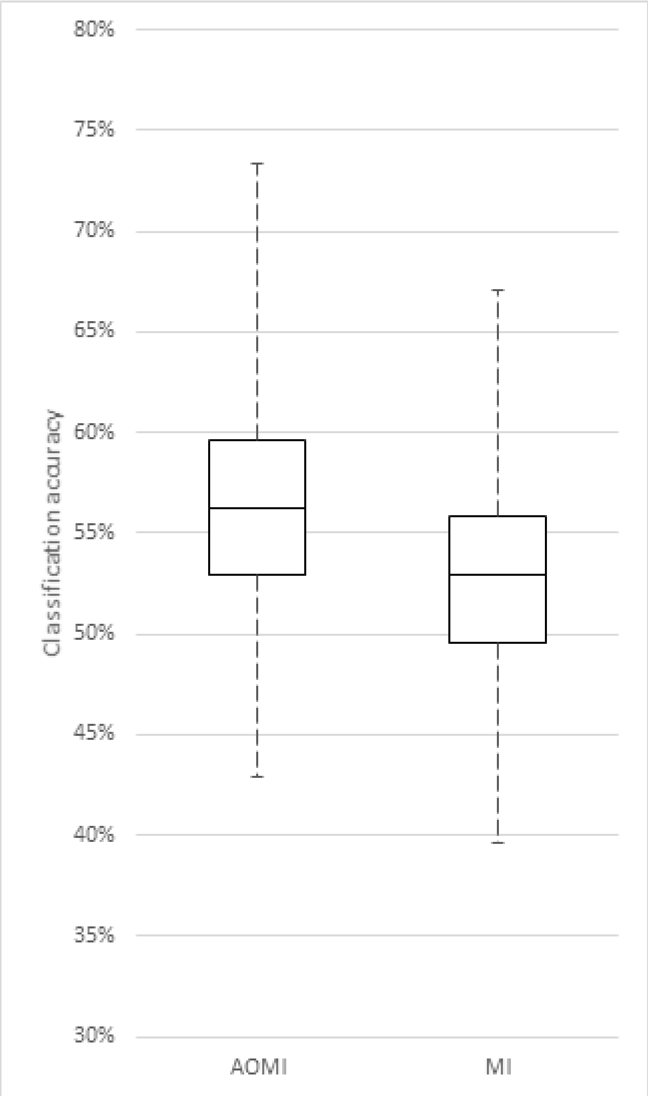

Figure S1. A box-whisker plot of BSC based on Hyperalignment of 2000 permutations of subject order showing the classification accuracies of different orders of subjects using AOMI and MI data of session 2.

2. Effect of subject order on classifier accuracy

To check whether the variation in Hyperalignment performance might be related to simply the first participant(s) used, we examined the participant orders used to obtain the low, middle and best classification results for the MI data. This is shown below in Figure S2, with three panels corresponding to sets of results at low, middle and high performance, where the y axis is classifier performance obtained for the subject order (left panel low classifier accuracy, right panel high classifier accuracy), the x axis is the subject order position and the colour scheme indicates subject number. No strong effect of subject order is evident, and accuracy appears independent of first subject entered into the algorithm.

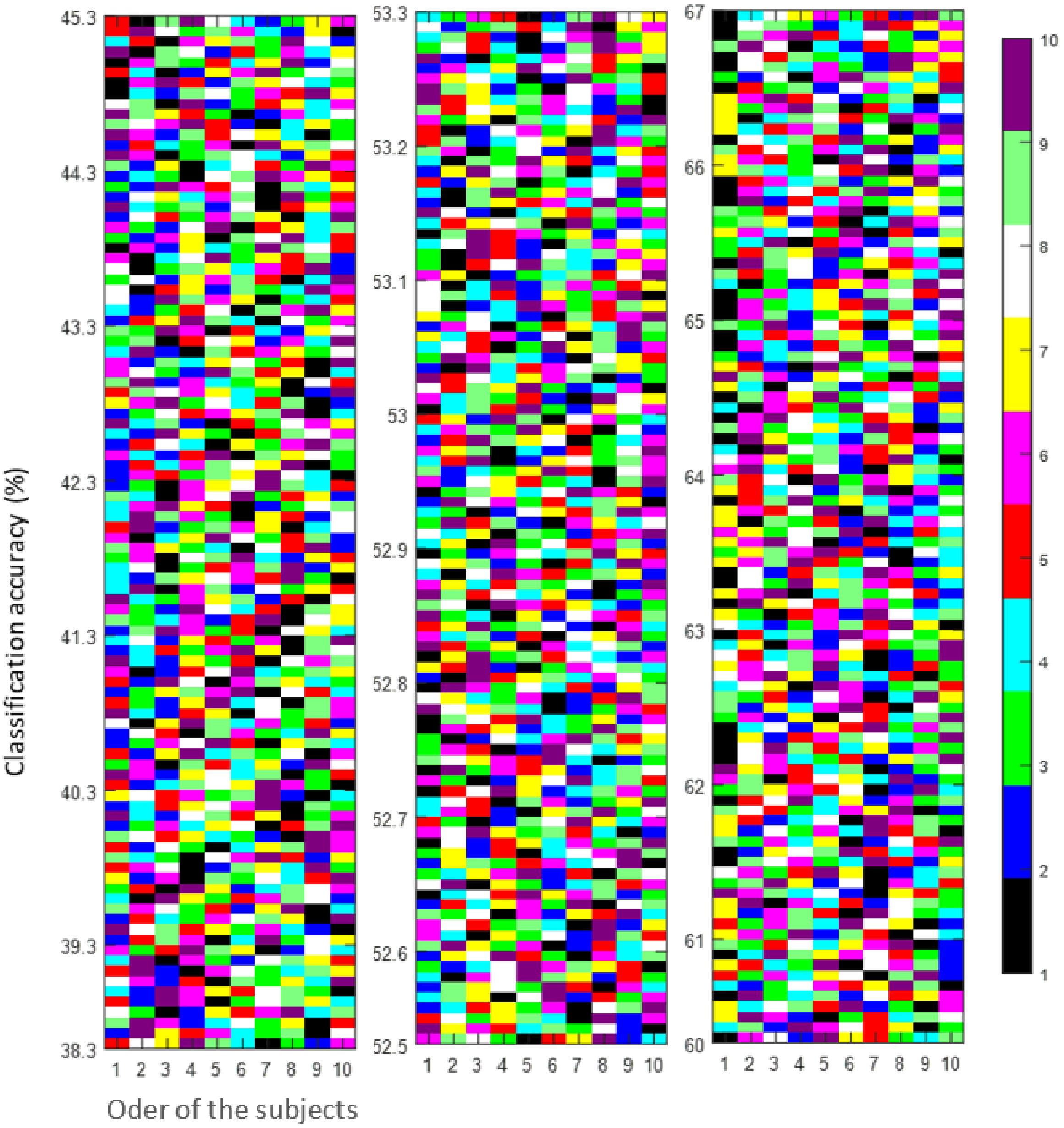

Figure S2. Illustration of the effect of subject order on classifier accuracy. Shown are three panels corresponding to low, middle and best results of classifier accuracy (shown on y axis). The ten colour coded items on each row show the subject order.

3. Between Subject Classification

Using the AOMI data of session 2, BSC based on anatomical alignment was 37.51% (SE=1.96%, chance=33%). In contrast, BSC based on Hyperalignment was 73.33% (SE=2.79%). The WSC accuracy was still below that of BSC based on Hyperalignment, as shown in Figure S3. In summary, our supplementary analyses confirm that Hyperaligment has a similar effect when applied to the MI and AOMI datasets. The trend for enhanced classification accuracies for the AOMI dataset is unsurprising, given that the Hyperalignment parameters were derived from an AOMI condition (Session 1).

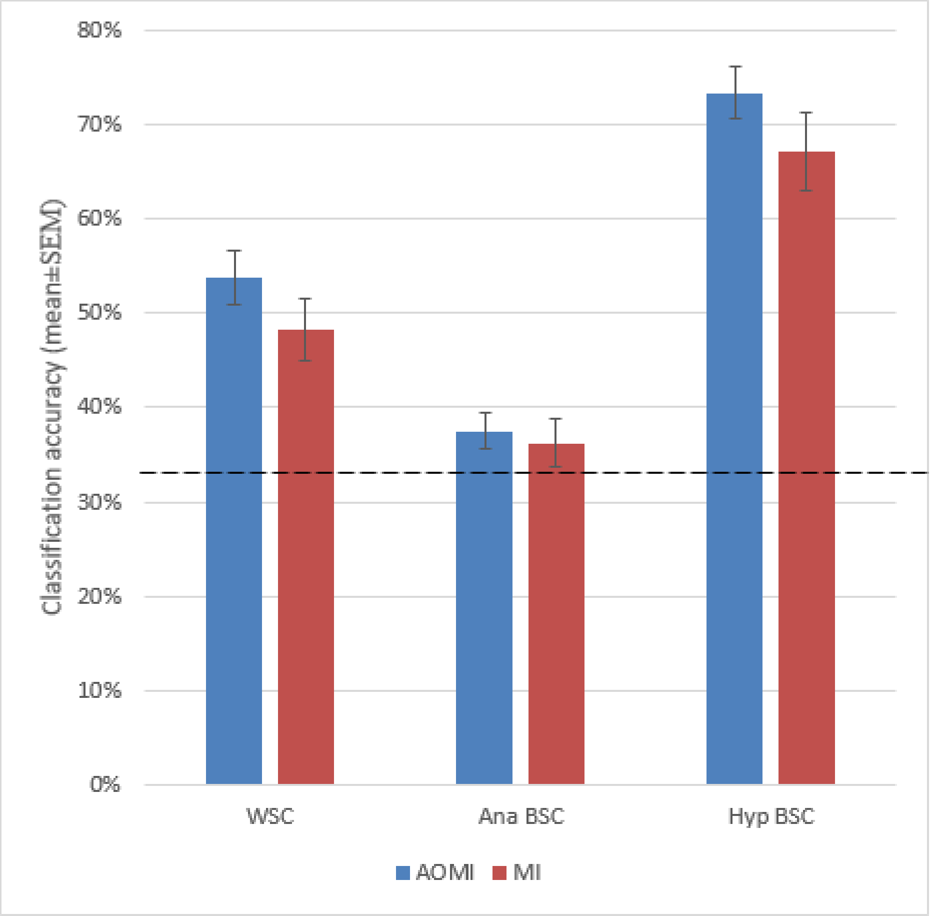

Figure S3. The classification accuracies (mean ±SEM) for averaged WSC, BSC based on Anatomical alignment and BSC based on Hyperalignment using AOMI and MI data of Session 2, dashed line indicates chance level (33%).

4. **Univariate analysis of Session 2.**

An exploratory univariate analysis was performed on the Session 2 data, in order to examine how brain activity varied between AO+MI and MI conditions as well as between each condition and baseline. For the first level analysis, all the pre-processed functional data of each subject were analysed using a General Linear Model (GLM) with six predictors (two modalities with three actions each), convolved with a hemodynamic response function. Group data were evaluated based on a second level random effect analysis general linear model (RFX-GLM). Three statistical maps were calculated which contrasting the AOMI vs baseline, MI vs baseline and AOMI vs MI respectively. These maps were corrected for multiple comparisons using FDR of 0.05.

The results are shown in Table S1. A single activation peak was found in the contrast MI > baseline. Reassuringly, this was coextensive with the peak for the supplementary motor area in the Searchlight map of BSC based on Hyperalignment shown in main Figure 5. The contrast AOMI > baseline mainly comprised activations in occipital cortex, and a small focus in the supplementary motor area was only sub-threshold in this contrast. These differences are backed-up by the direct contrasts between the two conditions.

Table 1, Clusters of brain activation for AOMI vs baseline, MI vs baseline and AOMI vs MI respectively, thersholded using FDR=0.05. (Note: x,y,z are given in Talairach 340 coordinates, LH= Left hemisphere. RH= right hemisphere. BA= Brodmann area.)

**Contrast**	**Cortical Area**	**BA**	**x**	**y**	**z**	***t***	***p*-Value**	**Size (1 mm Isotropic)**
**AOMI > MI**	RH, Inferior Temporal Gyrus	37	45	-67	-2	6.981	0.000038	55
	RH, Inferior Temporal Gyrus	19	46	-73	1	7.960	0.000012	69
	RH, Lateral Geniculum Body	-	24	-25	-2	7.337	0.000025	87
	LH, Inferior Occipital Gyrus	19	-42	-76	-2	15.387	<0.000001	405
**AOMI < MI**	RH, Superior Frontal Gyrus	6	9	-7	64	-7.718	0.000016	62
	RH, Medial Frontal Gyrus	6	6	-4	49	-7.284	0.000027	79
	RH, Medial Frontal Gyrus	6	3	-10	67	-9.094	0.000004	89
	LH, Cingulate Gyrus	24	-6	5	43	-7.515	0.00002	86
	LH, Claustrum	-	-33	5	10	-7.895	0.000013	166
	LH, Middle Frontal Gyrus	9	-39	36	37	-8.020	0.000012	66
**AOMI > baseline**	RH, Inferior Occipital Gyrus	18	45	-74	1	10.439	0.000001	598
	RH, Middle Occipital Gyrus	18	30	-91	7	5.801	0.000173	107
	LH, Lentiform Nucleus	-	-21	-1	13	6.314	0.000087	102
	LH, Inferior Occipital Gyrus	19	-42	-76	-2	7.563	0.000019	249
**MI > baseline**	LH, Medial Frontal Gyrus	6	-3	-10	61	8.941	0.000004	75
